# The new-look Lung India

**DOI:** 10.4103/0970-2113.45195

**Published:** 2009

**Authors:** S. K. Jindal

**Affiliations:** *Editor, Lung India*

With this issue of January 2009, Lung India has changed its looks and publication policy. The journal has entered into an agreement with M/S Medknow Publications, Mumbai for printing, publishing and distribution. This shall further improve the quality and the standard of the journal.

From now onwards, we adopt a complete on-line policy for submission of the articles, reviewing process, editing and proof- reading. All these facilities will be available at www.journalonweb.com/lungindia

This will save a lot of extra efforts and time for the authors, reviewers and editors. It might be a little difficult for some of the members in the beginning. But we are save that you will find the entire process as easy, quick and friendly. We solicit your cooperation for the success of this venture.

I, on behalf of the Editorial Board of Lung India invite you again to submit your articles and other interesting material to the journal. Your contribution is essential for the future of the journal.

Wishing you all a very happy and successful New Year.

